# Supplementary material for: Antioxidant vs. Prooxidant Properties of the Flavonoid, Kaempferol, in the Presence of Cu(II) Ions: A ROS-Scavenging Activity, Fenton Reaction and DNA Damage Study
Source: Int J Mol Sci. 2021 Feb 5;22(4):1619. doi: 10.3390/ijms22041619 (PMC7915082; doi:10.3390/ijms22041619)

Supplementary Figure S1: Radical scavenging activity of kaempferol and its Cu(II) complexes (1:1 and 1:2) using the ABTS assay. Time dependent decay of absorption band at 734 nm of $ABTS^{\bullet +}$ alone (black line), in the presence of kaempferol (red line), in the presence of kaempferol Cu complex (1:1) (blue line) and (1:2) (green line). The concentrations of kaempferol for Cu:kaempferol (1:1) complex and Cu:kaempferol (1:2) complex were 0.01 mM and 0.02 mM, respectively.

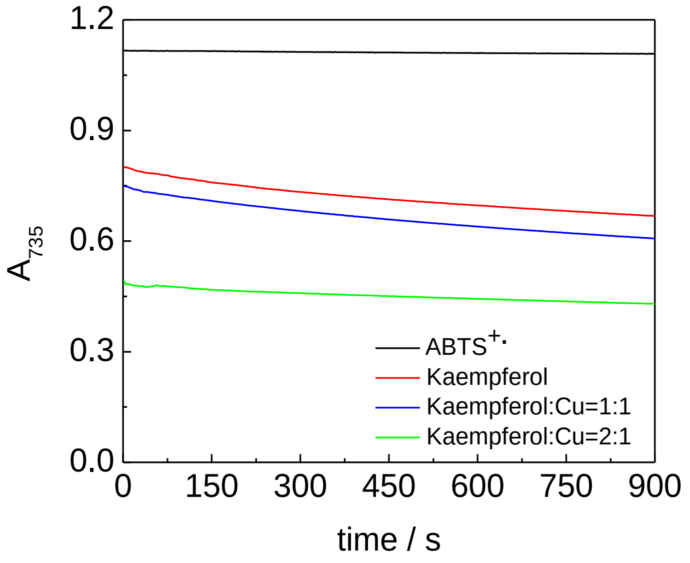

Supplement: Supplementary file 1 [file ijms-22-01619-s001.pdf]
